# Autografts vs Synthetics for Cruciate Ligament Reconstruction: A Systematic Review and Meta‐Analysis

**DOI:** 10.1111/os.12662

**Published:** 2020-03-16

**Authors:** Jian Sun, Xiao‐chun Wei, Lu Li, Xiao‐ming Cao, Kai Li, Li Guo, Jian‐gong Lu, Zhi‐qing Duan, Chuan Xiang, Lei Wei

**Affiliations:** ^1^ Department of Orthopaedics The Second Hospital of Shanxi Medical University; Shanxi Key Lab of Bone and Soft Tissue Injury Repair Taiyuan China; ^2^ The Institute of Stem Cell and Regenerative Medicine School of Medicine, Xiamen University Xiamen China; ^3^ Department of Orthopaedics Warren Alpert Medical School of Brown University/Rhode Island Hospital Providence Rhode Island USA

**Keywords:** Cruciate ligament, LARS, Meta‐analysis, Systematic review

## Abstract

To describe the outcomes of autografts and synthetics in anterior cruciate ligament (ACL) and posterior cruciate ligament (PCL) reconstruction with respect to instrumented laxity measurements, patient‐reported outcome scores, complications, and graft failure risk. We searched PubMed, Cochrane Library, and EMBASE for published randomized controlled trials (RCT) and case controlled trials (CCTs) to compare the outcomes of the autografts *versus* synthetics after cruciate ligament reconstruction. Data analyses were performed using Cochrane Collaboration RevMan 5.0. Nine studies were identified from the literature review. Of these studies, three studies compared the results of bone–patellar tendon–bone (BPTB) and ligament augmentation and reconstruction system (LARS), while six studies compared the results of four‐strand hamstring tendon graft (4SHG) and LARS. The comparative study showed no difference in Lysholm score and failure risk between autografts and synthetics. The combined results of the meta‐analysis indicated that there was a significantly lower rate of side‐to‐side difference > 3 mm (Odds Ratio [*OR*] 2.46, 95% confidence intervals [*CI*] 1.44–4.22, *P* = 0.001), overall IKDC (*OR* 0.40, 95% *CI* 0.19–0.83, *P* = 0.01), complications (*OR* 2.54, 95% *CI* 1.26–5.14, *P* = 0.009), and Tegner score (*OR* −0.31, 95% *CI* −0.52–0.10, *P* = 0.004) in the synthetics group than in the autografts group. This systematic review comparing long‐term outcomes after cruciate ligament reconstruction with either autograft or synthetics suggests no significant differences in failure risk. Autografts were inferior to synthetics with respect to restoring knee joint stability and patient‐reported outcome scores, and were also associated with more postoperative complications.

## Introduction

Arthroscopically assisted anterior cruciate ligament (ACL) or posterior cruciate ligament (PCL) reconstruction has been widely used for patients with cruciate ligament lesions, and advances in arthroscopic surgery have yielded good clinical results. However, controversy continues over the choice of graft tissue, including autografts, allografts, and synthetic ligaments. Two of the most common autografts used are bone–patellar tendon–bone (BPTB) and four‐strand hamstring tendon (4SHT)^1^. Arguably, autograft cruciate reconstruction is the gold standard, providing reliable long‐term results. Regardless of its type, autograft harvest can result in a degree of morbidity, which may negatively affect recovery after ACL reconstruction^2^. The use of allografts has increased in recent years because they offer less donor‐site morbidity, shorter surgical and anesthesia times, fewer postoperative complications, faster postoperative recovery, lower incidence of postoperative arthrofibrosis, less postoperative pain, and an unlimited graft source in the setting of multi‐ligament and revision reconstructions^3^, ^4^. However, allografts are associated with a higher expense, a risk of disease transmission, delayed healing, ligamentization, and an increased risk of graft rupture in the younger, more active population^4^, ^5^.

There have been numerous systematic reviews comparing the results of autograft hamstring *versus* bone–patellar tendon–bone grafts^6^, ^7^, ^8^, ^9^, ^10^. Data from this analysis suggested that there was insufficient evidence to recommend one graft choice over the other in major clinical results between graft types. Compared with 4SHT autografts, ACL reconstruction with BPTB autografts may more effectively restore knee joint stability and allow patients to return to higher levels of activity, but it may also result in an increase in long‐term anterior knee pain, kneeling pain, and higher rates of osteoarthritis.

There have also been many studies comparing autograft with allograft^5^, ^11^, ^12^. Prodromos *et al*.^11^ showed that the overall stability rate was 72% for all autografts compared with 59% for all allografts (*P* < 0.001), which did not account for the effect of irradiation on the allograft tissue. Christopher *et al*.^5^ and Mariscalco *et al*.^12^ compared exclusively nonirradiated allograft tissue with autograft in a systematic review and concluded that there was no significant difference between the two graft sources in outcomes.

The purpose of this systematic review was to conduct a meta‐analysis to compare the effectiveness of cruciate ligament reconstruction using either autografts or synthetics. We hypothesize that autografts or synthetics show no difference in: (i) instrumented laxity measurements; (ii) patient‐reported outcome scores; (iii) complications; or (iv) graft failure risk after cruciate ligament reconstruction.

## Materials and Methods

### *Literature Search and Study Selection*

The PRISMA (Preferred Reporting Items for Systematic Reviews and Meta‐Analyses) guidelines were followed from the inception of the study. A review of the literature was performed by two authors (L Li and XM Cao) using PubMed, Cochrane Library, and EMBASE from inception through January 2018. Key search terms included (“ACL,” OR “anterior cruciate ligament,” OR “PCL,” OR “posterior cruciate ligament,”) and [(“The Ligament Augmentation and Reconstruction System,” OR “LARS artificial ligament,” OR “artificial ligament”). No language limitations were imposed. The reference lists of the selected articles were reviewed to identify additional studies not found in the original search.

### *Inclusion and Exclusion Criteria*

Two researchers reviewed the generated list of unique articles for studies that met the following inclusion criteria: (i) Participants: the autografts and synthetics in the treatment of cruciate ligament lesions and minimum 2‐year follow‐up; (ii) Interventions and comparisons: at least two study groups in ACL or PCL reconstruction with either autograft or synthetics, the type of graft fixation was not limited; (iii) Outcome measure: the outcome assessments included instrumented laxity measurements, patient‐reported outcome scores, complications, and graft failure risk; (iv) Study design: CCTs, both randomized controlled trial (RCTs) and case controlled trials (CCTs). The following studies were excluded: (i) case‐based reports or reviews without original data; (ii) studies with imprecise experimental design; and (iii) studies with incomplete data or incorrect data.

### *Data Extraction and Quality Assessment*

Two investigators independently extracted data that met our inclusion and exclusion criteria. Disagreement between two reviewers was resolved by discussion or consultation with a third reviewer when necessary. The following information was extracted: details on study designs; patient demographic characteristics; length of clinical follow‐up, percentage lost to follow‐up, description of surgical technique, and clinical variables including follow‐up time, complications, knee laxity measurements with KT‐1000/KT‐2000 arthrometer, patient‐reported quantitative outcome measures (International Documentation Knee Documentation Committee [IKDC] grade, Lysholm score, Tegner activity scale). If a study did not specifically state whether it was retrospective or prospective in nature, it was assumed to be retrospective. Failure was defined either as complete or partial rupture of the ligament or as a documented “failure” as defined by each included study. The methodological quality of RCTs was evaluated independently by two of the authors (K Li and L Guo) using the tool for assessing risk of bias described in Chapter 8 of the Cochrane Handbook Systematic Reviews of Interventions (Version 5.1.0). This tool is comprised of a description and judgment for each entry in a “risk of bias” table, where each entry addresses a specific feature of the study. The judgment for each entry involves answering a question, with answers “Yes” indicating low risk of bias, “No” indicating high risk of bias, and “Unclear” indicating either lack of information or uncertainty over the potential for a bias. Quality assessment of non‐RCTs was performed according to the Methodological Index for Non‐Randomized studies (MINORs)^13^, which scores range from 0 to 24. Disagreement was resolved by consulting a third reviewer.

### *Statistical Analysis*

Data analysis was performed using RevMan 5.3 software. Two authors (JG Lu and ZZ Duan) checked the data during entry to ensure that there were no errors. Dichotomous outcomes were expressed in odds ratio (*OR*) and the mean difference (*MD*) was used for continuous outcomes, both 95% confidence intervals (*CI*). A χ^2^ test was used to assess heterogeneity between different studies, inter‐study heterogeneity was assumed in cases in which I^2^ > 50% or *P* value <0.1^14^, and *OR*s were pooled according to random‐effects models. Alternatively, fixed‐effects models were used. A *P* value less than 0.05 was considered statistically significant. A sensitivity analysis was conducted by excluding one study in each round and investigating the influence of a single study on the overall meta‐analysis estimate.

## Results

### *Search Results*

**Figure 1:**
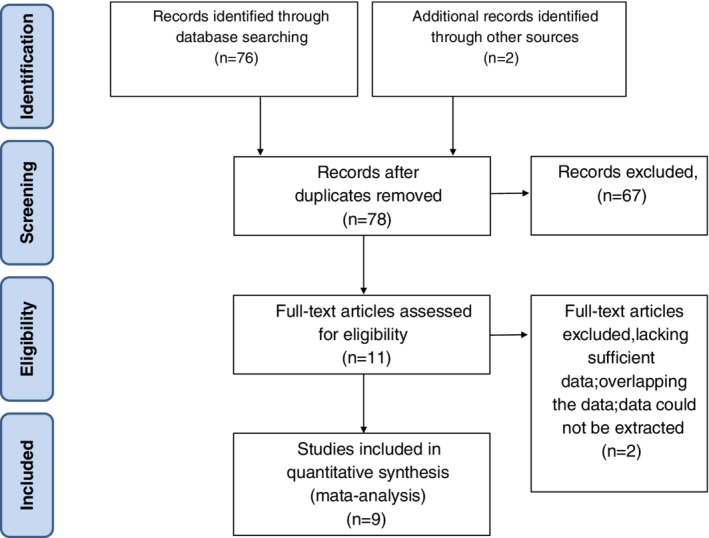
Flow chart of study selection process for the meta‐analysis.

A total of 56 potentially relevant articles were identified, with a review of article reference lists revealing a further two publications. After screening the titles and abstracts, 48 studies were excluded. After reading the full‐text of the remained 11 studies, we enrolled nine studies on 521 patients that met all inclusion criteria^2^, ^15^, ^16^, ^17^, ^18^, ^19^, ^20^, ^21^, ^22^, including seven English and two Chinese articles. The literature search process is presented in Fig. 1.

### *Study Characteristics*

The nine studies were published between 2002 to 2017 and comprised a total of 521 patients with an age range from 17 to 56 years. All studies’ reported groups were matched in terms of age, gender, severity, and course of disease. The mean length of follow‐up was 48.8 months (range, 24 to 122 months) in the allograft group and 48 months (range, 18 to 120 months) in the synthetics group. The allograft group contained 79.4% male and 20.6% female patients, and the synthetics group contained 76.5% male and 23.5% female patients. Of the nine studies included in this review, there were three randomized controlled trial, the other six studies were relatively high‐quality retrospective study (Table 1). One study used LARS as an augmentation to short or thin hamstring autograft rather than a sole constituent of the graft^2^.

**Table 1:** Overview of included studies*

Authors	Year	Journal	Procedure Date Range	Ligament	Patients enrolled (A/S)	Study Design	Country	Mean Length of Follow up (Range) months	% Follow‐up	Male/Female	Age,y, Mean (Range) (A/S)
Nau^15^	2002	JBJS	1996–1998	ACL	27/26	RCT	USA	24	96.23	36/17	30.87 ± 8.66/31.03 ± 8.98
Fan^16^	2008	CJRRS	2002–2005	ACL	27/15	Cohort	China	26.3(2243)/24.1(18–40)	100	34/8	20–52/17–40
Li^17^	2009	SICOT	2002–2006	PCL	15/21	RCT	China	28.8/26.4	100	30/6	20–43/18–47
Liu^18^	2010	SICOT	2003–2004	ACL	32/28	Cohort	China	49 (48–52)	100	45/15	32 (20–56)/36 (18–54)
Li 19	2012	EJOST	2007–2011	ACL	26/24	RCT	China	30	100	38/12	30.6(18–50)/32.6(18–50)
Pan^20^	2013	AOTS	2004–2006	ACL	30/32	Cohort	China	>48	100	44/18	33.93 ± 6.34/35.88 ± 11.30
Xu^21^	2014	AOTS	2006–2008	PCL	16/19	Cohort	China	51(46–57)	100	17/18	29.1 ± 5.7/ 28.6 ± 6.8
Hamido^2^	2015	OTSR	2007–2008	ACL	45/27	Cohort	Kuwait	59(58–62)	100	71/1	20(18–31)/24(21–35)
Chen^22^	2017	AJSM	2004–2007	ACL	73/38	Cohort	China	122.9 ± 11.8/120.8 ± 26.9	83	92/19	28.6 ± 8.8(17–52) /27.6 ± 9.3 (17–54)

* A/S, Autografts/Synthetics; ACL, anterior cruciate ligament; AJSM, The American Journal of Sports Medicine; AOTS, Arch Orthop Trauma Surg; CJRRS, Chinese Journal of Reparative and Reconstructive Surgery; EJOST, Eur J Orthop Surg Traumatol; JBJS, The journal of bone and joint surgery; OTSR, Orthopedics & Traumatology: Surgery & Research; PCL, posterior cruciate ligament; SICOT, International Orthopedics.

### *Assessment of Risk of Bias*

**Figure 2:**
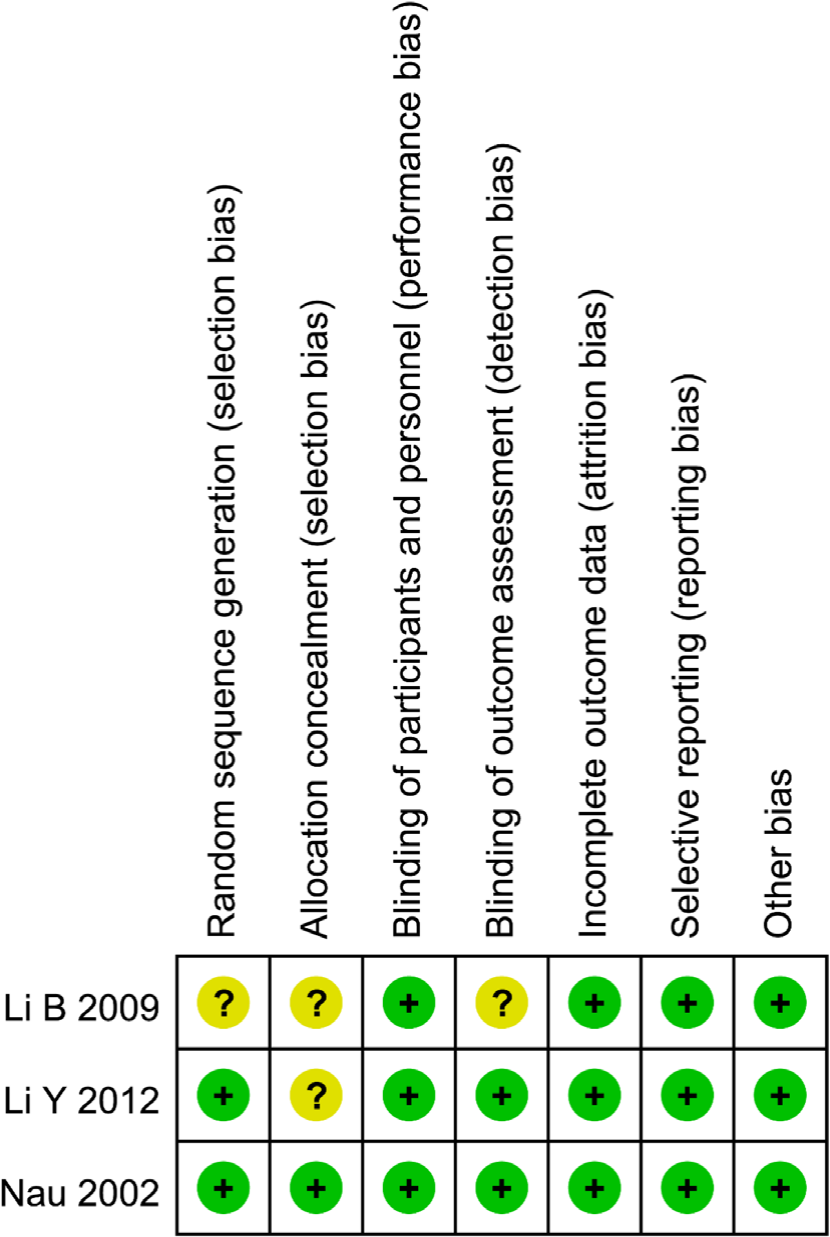
Quality of methodology of the RCTs. +, low risk of bias?, unclear risk of bias.

Three RCTs and six high‐quality retrospective studies included in this meta‐analysis have gone through a strict quality assessment. The Cochrane Handbook for Systematic Review of Interventions was consulted to assess the quality of RCTs (Fig. 2) For the three RCTs, all studies have definite selection criteria and are described as “randomized,” and two studies describe the methods for random sequence generation. One study describes allocation concealment or blinding methods^15^. All studies had a low risk of incomplete outcome data and selectively reporting results. The MINORS scale was applied for six non‐RCTs and the total score is 19.

### *Surgical Technique*

**Figure 3:**
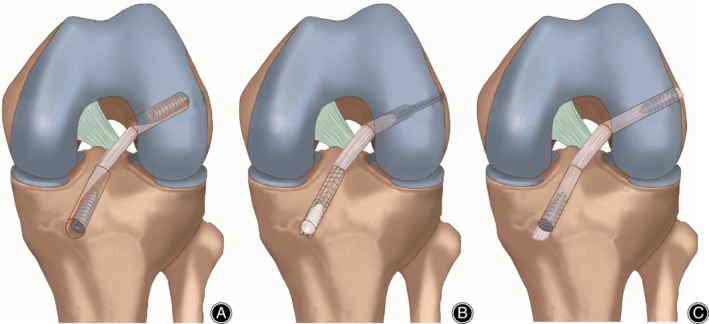
Schematic illustration of ACL reconstruction with BPTB(A), 4SHT(B) and LARS (C). BPTB, bone‐patellar tendon‐bone; 4SHG, four‐strand hamstring tendon graft; LARS, ligament augmentation and reconstruction system.

All surgeries were performed with the arthroscopic technique by the senior surgeon. Table 2 details the surgical techniques used for each report. For autograft procedures, three studies used BPTB and six studies used 4SHT (Fig. 3 A,B). For synthetic procedures, all studies used LARS (Fig. 3C). All studies used interference screw fixation for both tibial and femoral bone plugs in synthetic group, but most did not delineate whether these were metal or biocomposite, and only one study used bioabsorbable screw. Autograft fixation was slightly more variable, with femoral tunnels relying on interference screws in six studies^15^, ^17^, ^18^, ^19^, ^20^, ^21^, titanium button in two studies^16^, ^22^, bioabsorbable screw^2^ and crosspins in one study^22^. The tibial fixation of autografts included interference screws in six studies^15^, ^17^, ^18^, ^19^, ^20^, ^21^, bioabsorbable screw in one study^2^, steel plate in one study^16^, and screw and/or spiked washer in one study^22^.

**Table 2:** Overview of surgical details for included studies*

Authors	No. of Autografts (%)	No. of Synthetics (%)	Autografts	Synthetic	Autografts		Synthetics	
					Femoral Fixation	Tibial Fixation	Femoral Fixation	Tibial Fixation
Nau^15^	27	26	BPTB	LARS	inteinterference screw	interference screw	interference screw	interference screw
Fan^16^	27	15	4SHG	LARS	titanium button	steel plate	interference screw	interference screw
Li^17^	15	21	4SHG	LARS	inteinterference screw	interference screw	interference screw	interference screw
Liu^18^	32	28	4SHG	LARS	inteinterference screw	interference screw	interference screw	interference screw
Li^19^	26	24	BPTB	LARS	inteinterference screw	interference screw	interference screw	interference screw
Pan^20^	30	32	BPTB	LARS	inteinterference screw	interference screw	interference screw	interference screw
Xu^21^	16	19	4SHG	LARS	inteinterference screw	interference screw	interference screw	interference screw
Hamido^2^	45	27	4SHG	LARS	bioabsorbable screw	bioabsorbable screw	bioabsorbable screw	bioabsorbable screw
Chen^22^	73	38	4SHG	LARS	titanium button or; cross‐pin system	Screw and/or spiked washer	interference screw	interference screw

* BPTB, bone–patellar tendon–bone; LARS, ligament augmentation and reconstruction system; 4SHG, four‐strand hamstring tendon graft.

### *Instrumented Laxity Across All Studies*

**Figure 4:**
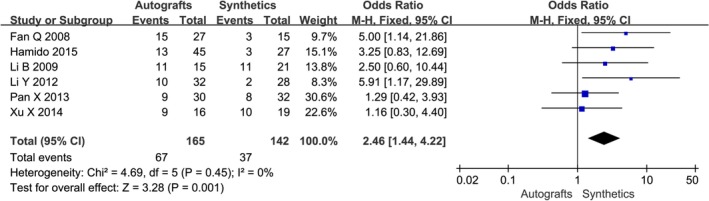
The forest plot of instrumented laxity autografts and synthetics after cruciate ligament reconstruction. In this and subsequent figures, CI, confidence interval; df, degrees of freedom.

Instrumented laxity testing was reported in eight studies^2^, ^16^, ^18^, ^19^, ^20^, ^21^, ^22^ as mean side‐to‐side difference at maximum follow‐up using maximum manual tension with either the KT‐1000 or KT‐2000 arthrometer (Table 3). The stability results of five studies showed that there was no significant difference between the two groups, four studies showed that the LARS group had significantly less anterior displacement than the autografts group. The pooled risk of instrumented anteroposterior laxity greater than 3 mm was 4.4% (95% *CI*, 0.6% to 11.6%) in the autografts group and 4.9% (95% *CI*, 2.8% to 7.4%) in the synthetics group (Fig. 4).

**Table 3:** Instrumented laxity*

Authors	Side‐to‐Side Difference Autografts, mm	Side‐to‐Side Difference Synthetics, mm	Significance
Nau^15^	2.38 ± 1.80	4.86 ± 3.80	ns
Fan^16^	5 (19%) > 3 mm	3 (20%) > 3 mm	ns
Li^17^	NR	NR	*P* < 0.05
Liu^18^	2.4 ± 0.5	1.2 ± 0.3	*P* = 0.013
Li^19^	8. 9 ± 4.2	5.3 ± 4.1	*P* = 0.004
Pan^20^	2.62 ± 2.12	2.29 ± 2.03	*P* > 0.5
Xu^21^	3.28 ± 1.95	3.27 ± 2.13	*P* > 0.05
Hamido^2^	2.5 ± 0.5	1.1 ± 0.3	*P* = 0.027
Chen^22^	2.4 ± 2.1	1.5 ± 1.5	*P* = 0.131

* Results are reported as mean ± SD. NR, not reported; ns, not significant.

### *Clinical Outcomes*

**Figure 5:**
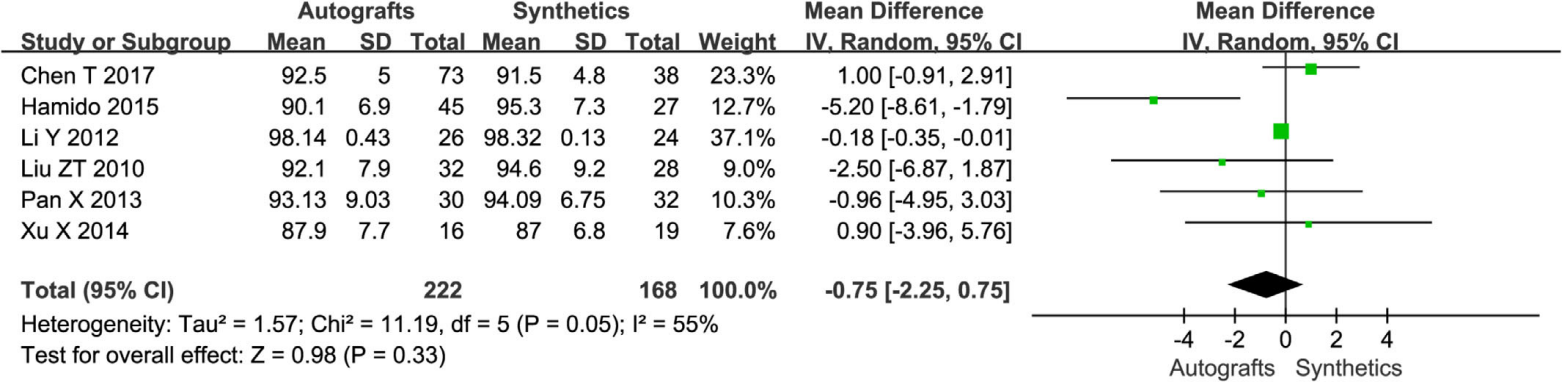
The forest plot of Lysholm score between autografts and synthetics after cruciate ligament reconstruction.

**Figure 6:**
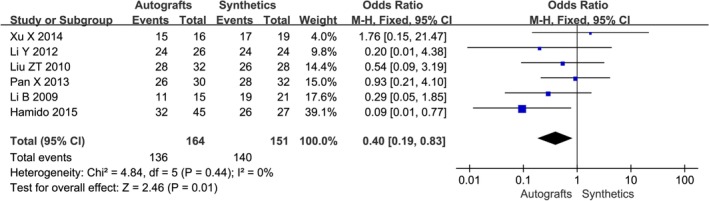
The forest plot of overall IKDC between autografts and synthetics after cruciate ligament reconstruction.

**Figure 7:**
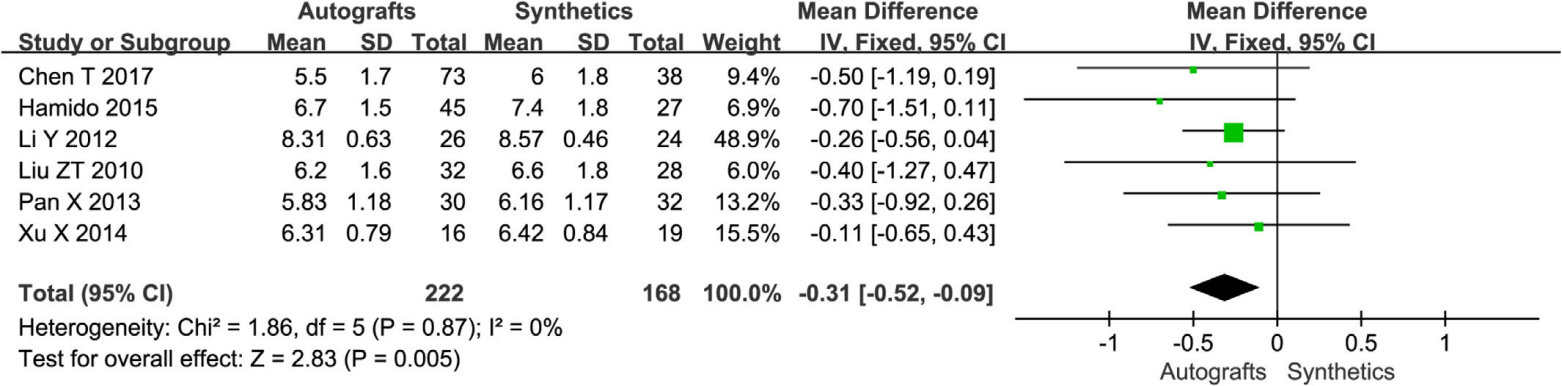
The forest plot of Tegner score between autografts and synthetics after cruciate ligament reconstruction.

A combination of IKDC scores, patient‐reported Lysholm scores, and/or Tegner activity scores were reported in all studies; the related information is detailed in Table 4. Six studies used the objective IKDC scoring system to describe the results of the ACL reconstruction as normal (A), nearly normal (B), abnormal (C), and severely abnormal (D). The IKDC scores of the six trials were treated as dichotomous variables: normal and nearly normal *versus* abnormal and severely abnormal. The IKDC scores were normal and nearly normal in 136/164 patients in the autografts group and 140/151 patients in the synthetics group, respectively. Mean Lysholm scores at follow‐up ranged from 85 to 98 in the autografts group and from 87 to 98 in the synthetics group. Mean Tegner scores at follow‐up ranged from 4.93 to 8.31 in the autografts group and from 5.03 to 8.57 in the synthetics group. The meta‐analysis showed no difference in Lysholm score after cruciate ligament reconstruction with autografts compared with synthetics (*OR* −0.75, 95% *CI* −2.25–0.75, *P* = 0.41) (Fig. 5). The combined results of the meta‐analysis indicated there was a significantly lower rate of overall IKDC (*OR* 0.40, 95% *CI* 0.19–0.83, *P* = 0.01) (Fig. 6) and Tegner score (*OR* −0.31, 95% *CI* −0.52–0.09, *P* = 0.005) (Fig. 7) in the autografts group than in the synthetics group.

**Table 4:** Clinical outcomes*

Authors	Overall IKDC			Lysholm			Tegner		
	Autografts	Synthetics	Significance	Autografts	Synthetics	Significance	Autografts	Synthetics	Significance
Nau^15^	NR	NR	ns	NR	NR	NR	NR	NR	ns
Fan^16^	NR	NR	NR	87.80 ± 3.41	88.90 ± 3.30	ns	4.93 (3–6)	5.03(3–7)	*P* > 0.05
Li^17^	8A,3B,3C,1D	14A,5B,2C,0D	*P* = 0.285	85 (33–100)	93 (43–100)	*P* < 0.05	6 (1–9)	7 (2–10)	*P* < 0.05
Liu^18^	22A,6B,4C,0D	21A,5B,2C,0D	*P* > 0.05	92.1 ± 7.9	94.6 ± 9.2	*P* = 0.259	6.2 ± 1.6	6.6 ± 1.8	*P* = 0.387
Li^19^	22A,2B,2C,0D	22A,2B,0C,0D	*P* = 0.448	98.14 ± 0 0.43	98.32 ± 0.13	*P* = 0.055	8.31 ± 0.63	8.57 ± 0.46	*P* = 0.105
Pan^20^	14A,12B,4C,0D	19A,9B,4C,0D	*P* > 0.1	93.13 ± 9.03	94.09 ± 6.75	*P* > 0.5	5.83 ± 1.18	6.16 ± 1.17	*P* > 0.05
Xu^21^	9A,6B,1C,0D	10A,7B,2C,0D	*P* > 0.05	87.9 ± 7.7	87.0 ± 6.8	*P* > 0.05	6.31 ± 0.79	6.42 ± 0.84	*P* > 0.05
Hamido^2^	26A,6B,11C,2D	20A,6B,1C,0D	*P* = 0.05	90.1 ± 6.9	95.3 ± 7.3	*P* = 0.239	6.7 ± 1.5	7.4 ± 1.8	*P* = 0.368
Chen^22^	91.6 ± 5.1	89.8 ± 5.3	*P* = 0.124	92.5 ± 5.0	91.5 ± 4.8	*P* = 0.251	5.5 ± 1.7	6.0 ± 1.8	*P* = 0.286

* IKDC, International Knee Documentation Committee; NR, not reported; ns, not significant.

### *Complications*

**Figure 8:**
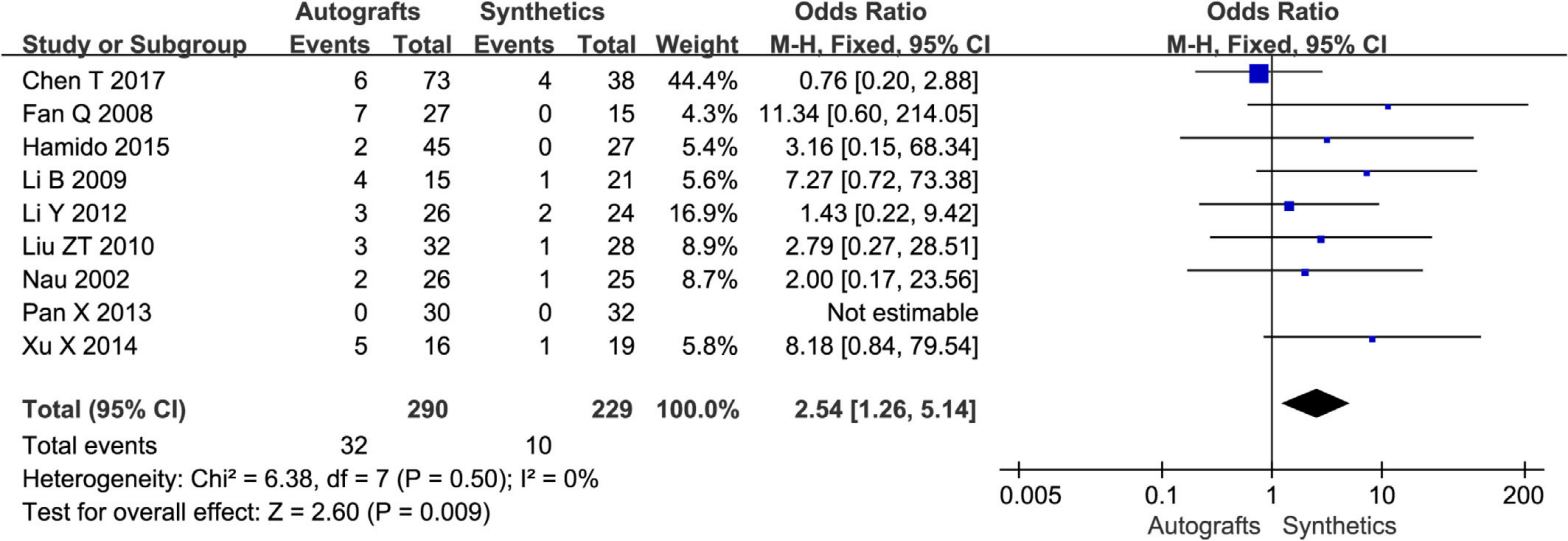
The forest plot of complications between autografts and synthetics after cruciate ligament reconstruction.

Table 5 details the complications encountered in each group. The complications results of eight studies showed that there was no significant difference between the two groups and just one study^16^ showed that the synthetics group had significantly less complications than the autografts group. Table 5 details the complications encountered in each group at the time of most recent follow‐up. The meta‐analysis results are shown in Fig. 8. There was a statistical difference among the postoperative complications between the two groups (OR 2.54, 95% CI 1.26–5.14, *P* = 0.009), indicating that complications of synthetics were less than autografts.

**Table 5:** Complications*

Authors	Autografts			Synthetics			
	No. of Patients at Follow‐up	Complications,% (n)	Description of Complications	No. of Patients at Follow‐up	Complications,% (n)	Description of Complications	Significance
Nau^15^	26	7.7 (2)	1 Superficial infection; 1 Screw‐related problem	25	4 (1)	1 Screw‐related problem	ns
Fan^16^	27	25.9 (7)	2 loss of last 5° of extension; 5 loss of 5–10° of full flexion	15	0 (0)	NR	*P* < 0.05
Li^17^	15	26.7 (4)	1 anterior knee Pain; 2 paraesthesia; 1 arthrofibrosis	21	4.8 (1)	1 anterior knee pain	NR
Liu^18^	32	9.4 (3)	2 loss of 5° of full flexion; 1 arthrofibrosis	28	3.6 (1)	1 Screw‐related problem	NR
Li^19^	26	11.5 (3)	1 Screw‐related problem 2 anterior knee	24	8.3 (2)	1 Screw‐related problem; 1 anterior knee Pain	NR
Pan^20^	30	0 (0)	NO	32	0 (0)	NO	NR
Xu^21^	16	31.3 (5)	2 anteromedial knee pain; 3 paraesthesia	19	5.3 (1)	1 synovitis	NR
Hamido^2^	45	4.5 (2)	1 paraesthesia; 1 arthrofibrosis and loss of; last 5–10° of extension	27	0(0)	NR	NR
Chen^22^	73	8.2 (6)	2 Screw‐related problem; 3 Donor site morbidity, 1Superficial infection	38	10.5 (4)	3 Screw‐related problem; 1Synovitis	NR

* NR, not reported; ns, not significant.

### *Failure Risk Across All Studies*

Graft rupture was labeled as failure, with other reasons for reoperation categorized as “complications.” Nau *et al*.^15^ reported two patients were lost to follow‐up (one in each group) and each was assumed to be a failure of reconstruction. Chen *et al*.^22^ evaluated primary ACL reconstruction using either synthetics with remnant preservation or hamstring autografts and observed cumulative failure in three patients (3/38, 7.9%) and six patients (6/73, 8.2%), respectively, for patients with synthetics and hamstring autografts at 10 years post‐operation. Seven studies reported zero failures. No studies showed any statistically significant difference in the rates of graft failure between the two groups.

## Discussion

A substantial body of literature has examined the factors influencing outcome after ACL reconstruction, including comparisons of tunnel placement, fixation technique, and graft choice. Although many studies argue in favor of one form of autograft over another, confounding variables of such a complex surgery are often difficult to control, and may influence results^9^. Meta‐analysis allows us to quantitatively analyze multiple prospective comparative studies with similar study objectives to increase sample size and improve statistical power. Thus, we performed this up‐to‐date meta‐analysis of comparative studies to assess the safety and efficacy of cruciate ligament reconstruction that use either autografts or synthetics in order to provide a reference for the selection of autograft. Our study had the advantage of long‐term follow‐up of more than 2 years and is the first study to our knowledge comparing these two grafts.

Admittedly, autograft cruciate reconstruction is the gold standard, providing reliable long‐term results, while this method somewhat reminds us of robbing “Peter to pay Paul.” Logically, an off‐the‐shelf graft without self‐tissue sacrifice would be an ideal choice. Shorter surgical and anesthesia time, fewer postoperative complications, reduced morbidity at the harvest site, faster postoperative recovery and lower incidence of postoperative arthrofibrosis, and less postoperative pain are considered to be the main advantages of allograft usage for ACL replacement^3^. On the other hand, allograft usage may be associated with higher rates of re‐rupture, limited availability, a delayed healing and ligamentization process in comparison to autografts, risk of disease transmission, and expensiveness^23^. Since the 1970s, synthetic devices have been available for use in the management of the cruciate‐injured knee. These devices have the intended benefits of avoiding donor‐site morbidity, providing a strong stabilizing construct, and allowing aggressive rehabilitation and a relatively rapid return to sporting activity without the risks of disease transmission and rejection. LARS is a third generation of such synthetic ligament, designed to overcome the issues of graft failure and synovitis which led previous generations of synthetic ligaments to fall out of favor. It consists of two distinct segments: an intraosseous and an intra‐articular segment. Its intraosseous segment is composed of longitudinal fibers bound together by a transverse knitted structure while the intra‐articular segment consists of multiple parallel longitudinal fibers twisted at 90° angles^24^. The intra‐articular multifilament part of the prosthesis is implanted in a twisted fashion to imitate the natural cruciate ligament. This avoids shearing forces between the fibers and interferes during combined tension, torsion, and flexion. Additionally, the intra‐articular segment of the graft acts as a scaffold inducing fibroblastic ingrowth between the fibers of the ligament due to the porosity of the material^25^, ^26^, ^27^. Ingrown soft tissue between the ligament fibers acts as a viscoelastic element and protects the ligament against friction at the opening of the bony canal as well as between the artificial fibers themselves^15^.

Our meta‐analysis included nine studies. The pooled ORs of these studies indicated that autografts were inferior to synthetics for restoring knee joint stability, patient‐reported outcome scores and were associated with more postoperative complications, but there was no significant difference between two groups with regard to graft failure in any of the studies. Another drawback of autografts is that graft strength decreases during the long period of revascularization. This may lead to graft laxity or rupture during early rehabilitation^28^. Currently, the BPTB and hamstring autografts are the most commonly used grafts. The autologous and allogeneic bone grafts in the bone tunnel must undergo tendon “religamentization.” However, the phenomenon of ligamentization occurs in the successfully reconstructed human cruciate ligament between 6 month to 1 year after operation, being slowly revascularized and presenting most histologic and functional properties^28^, ^29^. In this process, necrosis and replacement of bone graft occurs alternatively, which is prone to collapse and loosening during this course. The tunnel wall is reconstructed by fiber tissue and the enlargement of the tunnel is likely to occur during this period, but the artificial ligament in the bone tunnel did not undergo necrosis or religamentization^30^. The synthetic materials can provide immediate strength and stability for the knee after reconstruction, allowing for early function recovery^18^, ^30^. Four studies evaluated patient‐reported outcomes postoperatively at different times of follow‐up, which suggested that symptom relief and restoration of function of those with synthetics were statistically higher at 6 months or 1 year postoperatively, demonstrating that patients with synthetics could return to sports earlier^15^, ^17^, ^19^, ^22^. Krupa *et al*. also indicated a significant progress from preoperative to short‐term postoperative result in reducing anterior translation and anterolateral rotational instability of the tibia relative to the femur in patients who had undergone ACL reconstruction with a synthetic LARS graft^4^.

The strengths of this review are its well‐conducted clinical trials and relatively long‐term follow‐up of the papers included in the analysis. To our knowledge, no previously published systematic review of the influence of autograft choice on outcome after cruciate ligament reconstruction surgery has had such rigid inclusion and exclusion criteria. The included studies are all high‐level data with at least two‐year minimum follow‐up and thus provide a high level evidence for clinical decisions. A review by Newman and Atkinson indicated the support of the current literature for the use of LARS in the short to medium term in patients who have undergone ACL reconstruction. However, the authors highlighted the need for high‐quality studies with long‐term follow‐up to determine whether the use of LARS is preferable to autologous grafts^31^. Batty *et al*. wrote systematic review was to assess the safety and efficacy of different synthetic devices in cruciate ligament surgery, a limitation of this review is the paucity of well‐conducted clinical trials included and the exclusion of no‐English‐language studies (*n* = 8)^32^. Another two systematic reviews assess the effectiveness of LARS as a surgical option for ACL and PCL, but they were limited by the paucity of high‐level, high‐quality evidence^33^, ^34^.

Be that as it may, there are several limitations in this study. Firstly, although nine studies were added in this study since the latest systematic review, just three of them were RCT, the other six studies were relatively high‐quality retrospective studies, and thus usually has more potential sources of bias and confounding factors. This might weaken the strength of the findings, and more high‐quality randomized controlled trials with long‐term follow‐up are necessary to make a firm conclusion. Secondly, all the articles included ACL and PCL reconstruction. Thirdly, the majority of studies were limited in their statistical power by small sample size. Further standardization of rehabilitation, utilization of a blinded clinical examiner, and use of additional validated patient‐reported outcome measure would improve the strength of conclusions and meta‐analysis.

Our study is the first meta‐analysis to compare the results of the autografts and synthetic ligaments. The combined results of this meta‐analysis indicate a statistical difference between autografts and synthetics in terms of negative outcomes of instrumented laxity, IKDC scores, patient‐reported Lysholm scores, and Tegner activity scores. Thus, our study concludes that cruciate ligament reconstruction with LARS achieved better postoperative effects in terms of restoring knee joint function and stability than autografts and was associated with less postoperative complications. The main advantages of the use of a synthetic ligament in ACL reconstruction are: the immediate recovery of stability and the timely rehabilitation and avoidance of sacrifice of autologous structures. We consider LARS artificial ligament to be an alternative graft for cruciate ligament reconstruction, especially (i) when early rehabilitation is imperative, (ii) in the presence of multiple ligament injuries, and (iii) in revision surgeries in which the availability of autologous tissue for reconstruction is limited.
